# Sociodemographic patterns of psychological distress, non-communicable diseases, and disability among south Africans 60 years and older: a generalized structural equation modelling approach

**DOI:** 10.3389/fragi.2026.1983741

**Published:** 2026-10-29

**Authors:** Sbonelo Chamane, Simangaliso Mnyandu, Lehlogonolo Makola, Musawenkosi Mabaso, Zaynab Essack, Ronel Sewpaul, Sean Jooste, Sizulu Moyo, Nompumelelo Zungu, Khangelani Zuma

**Affiliations:** 1 Public Health, Societies and Belonging Division, Human Sciences Research Council, Pretoria, South Africa; 2 School of Public Health, University of the Witwatersrand, Johannesburg, South Africa; 3 School of Law, College of Law and Management Studies, University of KwaZulu-Natal, Pietermaritzburg, South Africa; 4 School of Public Health, University of Cape Town, Cape Town, South Africa; 5 School of Nursing and Public Health, University of KwaZulu Natal, Howard College, Durban, South Africa

**Keywords:** ageing, disability, non-communicable diseases, psychological distress, socio-demographic factors, South Africa

## Abstract

**Background:**

South Africa is experiencing a steady increase in ageing population alongside a growing burden of non-communicable diseases (NCDs), disability and mental health challenges. However, the interplay between these conditions in later life remains underexplored.

**Methods:**

The study draws data from the sixth South African National HIV Prevalence, Incidence and Behaviour Survey which used a multi-stage, stratified, cluster random sampling design. The analysis estimated the prevalence of self-reported psychological distress, NCDs and disability among adults aged 60 years and older. Sociodemographic factors associated with these outcomes were examined using a Generalized Structural Equation Model.

**Results:**

Among adults aged 60 years and older, the prevalence of psychological distress was 3.1%, NCDs 59.7%, and disability 64.1%. Having at least one NCD significantly increased the likelihood of psychological distress, while having a disability increased the likelihood of having an NCD. Women and those aged 70–79 years had higher odds of both NCDs, and disability compared to men and those aged 60–69 years, respectively. Compared to Black Africans, participants from other racial groups had lower odds of distress but higher odds of NCDs. Relative to primary education, secondary education lowered the odds of distress and disability but increased the odds of NCD, while tertiary education was only protective against disability. Unemployment was associated with higher odds of NCDs, while residing in rural informal areas and never being married was associated with lower odds of NCDs only, compared to those who were employed, living in urban areas and ever married, respectively.

**Conclusion:**

The findings highlight strong interconnections between chronic disease, disability and mental health in later life, shaped by age, sex, education, marital status and place of residence. Interventions that integrate social, physical and psychological care are essential to improving healthy ageing outcomes in South Africa.

## Background

The 21st century is witnessing one of the most significant demographic transformations in human history characterised by a rapid global ageing population (Gu et al., 2021), driven by declining fertility rates and substantial increases in life expectancy (Aitken and Norman, 2025; Gu et al., 2021; WHO, 2025b). The proportion of older adults, particularly those aged 60 years and above, is growing at an unprecedented pace. According to the World Health Organization (WHO), the global population of people aged 60 and older is projected to more than double, rising from 1 billion in 2020 to over 2.1 billion by 2050 (WHO, 2025a). For the first time in human history, the proportion of older persons is overtaking youth, and growth is highly concentrated in a handful of lower- and middle-income nations, while regions like Europe and East Asia are experiencing steady population decline (Navaneetham and Arunachalam, 2025; WHO, 2025a). South Africa, is also experiencing accelerated aging as public health advances, including expanded roll-out of antiretroviral therapy (ART), are extending life expectancy despite ongoing socioeconomic and health system challenges (Bredenkamp et al., 2021; Solanki et al., 2019; Zuma et al., 2025). The country has shown notable recovery in recent decades with life expectancy increasing from a low of around 54 years in the early 2000s to approximately 65 years by 2023 (Doan et al., 2022). However, sex disparities persist, with males living an average of 62 years compared to 68 years for females, a reflection of both biological and social determinants of longevity (Carmel, 2019).

Evidence shows that there is usually a gap between lifespan (the years lived) and health span (years lived in good health) due to significant health and social challenges associated with increased life expectancy (Khan et al., 2024; WHO, 2020). Ageing is inherently associated with heightened vulnerability to chronic health conditions, functional limitations, and psychological strain (Jaul and Barron, 2017). Older populations are increasingly susceptible to chronic health conditions such as cardiovascular disease, diabetes, chronic respiratory issues, and cancer which are the leading causes of morbidity and mortality in this population group worldwide (Belayneh et al., 2024). Concurrently, age-related functional limitations and disabilities often emerge, further complicating health trajectories and diminishing quality of life (Khalili et al., 2024). Critical but often overlooked, is the psychological wellbeing of ageing, which encompasses aspects such as life satisfaction, emotional stability, purpose in life, and overall mental health (Trudel-Fitzgerald et al., 2019).

Consequently, globally the most pressing concerns for older populations are three interrelated dimensions of health and wellbeing: psychological distress (including stress, anxiety, and depressive symptoms), disability (defined as limitations in performing basic or instrumental activities of daily living), and the burden of NCDs such as hypertension, diabetes, stroke, chronic respiratory diseases, and cancer. These three elements do not operate in isolation; rather, they intersect and mutually reinforce one another in ways that can profoundly shape the ageing experience. For example, the poor management of an NCD like diabetes may lead to sight and mobility impairments, which in turn can trigger social isolation and psychological distress (Ida et al., 2020). Conversely, chronic stress and poor mental health can exacerbate the progression of NCDs and accelerate functional decline, creating a cyclical pattern of deteriorating health (Everard et al., 2025).

Recognizing these interdependencies, global and national health policies have increasingly emphasized integrated approaches to ageing and chronic disease management. The WHO’s Global Strategy and Action Plan on Ageing and Health (2016–2030) calls for person-centered, integrated care models that address multimorbidity, functional ability, and mental wellbeing simultaneously (Rudnicka et al., 2020). Similarly, South Africa’s National Policy on the Care and Support of Older Persons and its National Strategic Plan for the Prevention and Control of NCDs (2022–2027) underscore the need for coordinated services that ensure equitable access to care, reduce disability, and promote psychological resilience among older adults (Basu, 2022). Despite these policy commitments, implementation remains fragmented. Health systems often operate in silos; mental health services are often disconnected from chronic disease programs, and rehabilitation or disability support is rarely integrated into primary care for older people (Peiris et al., 2024). This disconnect limits the effectiveness of interventions and fails to address the synergistic nature of these health outcomes.

While policy frameworks outline the structural commitments to healthy aging, understanding the underlying determinants that shape health outcomes in later life remains equally critical. Moreover, the determinants of psychological wellbeing, disability, and NCDs are inherently multifaceted, shaped by an interplay of biological, behavioural, socioeconomic, and environmental factors (Rasesemola et al., 2023). Socioeconomic status, educational attainment, access to healthcare, social support networks, lifestyle behaviours (e.g., physical activity, diet, smoking), and neighbourhood characteristics all play critical roles in shaping health outcomes in later life (Hamasaki, 2025). However, the pathways through which these determinants operate and the ways they differentially or jointly influence psychological wellbeing, disability, and NCDs remain insufficiently understood. Advancing knowledge on these pathways is essential not only for refining theoretical models of aging and wellness but also for informing targeted, person-centered interventions that promote healthy and successful aging.

A critical gap persists in the empirical literature. Existing research has largely examined psychological distress, disability, and NCDs as separate domains of older-adult health. For instance, studies have explored links between chronic diseases and psychological distress, while others have investigated how common mental disorders and chronic conditions influence disability (Salazar et al., 2025; Xiao et al., 2022). Evidence from South Africa similarly remains fragmented. Studies among older South Africans have demonstrated associations between depression and functional disability, while research in rural South Africa has also examined the relationship between multimorbidity and physical and psychological functioning (Chang et al., 2019; Tomita and Burns, 2013). Similarly, some studies have explored the intersections between disability, chronic disease, and psychological distress in specific contexts, i.e., general population and in a conflict zone (Summers et al., 2019; Waterhouse et al., 2017). Nonetheless, few studies have jointly analysed these three dimensions within a unified analytical framework, particularly among adults aged 60 years and older in South Africa and other African settings. Most existing analyses treat these domains as separate outcomes or adjust for one while studying another, thereby overlooking the reciprocal pathways and shared determinants that link them. Therefore, this study aims to assess the burden and socio-demographic patterning of NCDs, psychological distress and disability among older South Africans, and to examine the social determinants and interrelationships of these outcomes using a Generalized Structural Equation Model.

## Methodology

This study used data from the sixth South African National HIV Prevalence, Incidence and Behaviour Survey which was a cross-sectional population-based stratified household survey conducted in 2022 (Zuma et al., 2025). A two-stage stratified cluster random sampling approach was applied. In the first stage, 1,000 small area layers (SALs) were stratified by province, locality type and race group. These were primary sampling units. To allow oversampling at district level, an additional 1,144 SALs were included, resulting in a total of 2,144 SALs. In each SAL a systematic random sample of 15 Visiting Points (VPs) was sampled, giving a total of 32 160 VPs, of which 27 005 (91.7%) were valid. Of these, 21 615 households participated, yielding a response rate of 80%. The ultimate sampling unit were all individuals living in the selected households. People living in institutions such as care homes, shelters, student residences or military barracks were excluded, as were individuals who could not provide informed consent. Data were collected electronically using tablets with four structured questionnaires tailored for (1) head of the household, (2) parents or guardians of children aged 0–11 years, (3) children aged 12–14 years and (4) individuals aged 15 years and older. The instruments collected household and age-appropriate data on socio-demographic factors, HIV related knowledge and behaviours, and health conditions. This study uses data from respondents aged 60 years and above, who responded to the health question related to psychological distress, non-communicable diseases, and disability.

## Ethical clearance

Ethical approval for the survey was granted by the Human Sciences Research Council (HSRC) Research Ethics Committee (REC) with protocol approval number REC:4/18/11/15 The U.S. Centers for Disease Control and Prevention (CDC) reviewed the protocol in accordance with human research protection procedures and relied on the local REC for approval^1^.

### Statistical measures

#### Endogenous variable

Psychological distress is increasingly recognised as an NCD, and in this study it was operationalised as a psychological NCD. Psychological distress was treated as the primary endogenous outcome and was measured using the Kessler Psychological Distress Scale (K10). This instrument captures non-specific symptoms of anxiety and depression based on respondents’ experiences over the previous 4 weeks. The K10 includes the following questions: feeling tired for no reason; feeling nervous; feeling so nervous nothing could calm you down; feeling hopeless; feeling restless or fidgety; feeling so restless you could not sit still; feeling depressed; feeling that everything was an effort; feeling so sad that nothing could cheer you up; and feeling worthless. A total score of 20 or above was considered indicative of psychological distress, while scores below 20 were classified as absence of distress. The full scale was retained to preserve the intended construct and established scoring approach. Although some items assess experiences such as tiredness and effort that may also occur in the context of chronic illness or functional limitations, these items contribute to the broader construct of psychological distress and were not considered sufficient grounds for excluding individual items from the scale. The resulting measure was interpreted as psychological distress rather than as a diagnosis of a specific mental disorder.

The secondary endogenous variable was physiological NCD status. All self-reported conditions including hypertension, diabetes, cancer, heart disease, other cardiovascular conditions, asthma, chronic lung disease, and chronic liver disease were considered, and individuals reporting at least one were classified as having a physiological NCD while those reporting none were classified as not having an NCD.

The third endogenous variable was disability, measured using the Washington Group Short Set on Functioning (WG-SS). This included six domains: difficulty seeing even with glasses, difficulty hearing even with a hearing aid, difficulty walking or climbing steps, difficulty remembering or concentrating, difficulty with self-care such as washing or dressing, and difficulty communicating using usual language. Response options for each domain were: 1 = No difficulty, 2 = Some difficulty, 3 = A lot of difficulty, 4 = Cannot do at all. Individuals reporting some difficulty or more in any domain or disability type were classified as having a disability or impairment, while those reporting no difficulty in all domains or disability type were classified as not having a disability. This operational definition captures a broad spectrum of functional limitations, including less severe difficulties, and is distinct from more restrictive WG-SS thresholds used to identify disability for other analytical purposes. The NCD variable was operationalised as a binary indicator of reported NCD status to examine its relationship with psychological distress and functional difficulties. This approach focused on the presence or absence of an NCD rather than the number of co-occurring conditions or condition-specific associations. Accordingly, the variable was not intended to measure multimorbidity, and the findings should be interpreted in relation to reported NCD status rather than the burden of individual diseases.

### Exogenous variable

In alignment with the study objectives and established demographic frameworks, the following exogenous variables were included to account for sociodemographic and contextual variation: Sex (Male, Female); Age (60–69, 70–79, 80+); Race group (Black African, Other races); Marital status (Ever married, Never married); Education level (Non/Primary, Secondary, Tertiary); Employment status (Employed, Unemployed); Locality type (Urban, Rural informal, Rural formal); Province (Western Cape, Eastern Cape, Northern Cape, Free State, KwaZulu-Natal, North West, Gauteng, Mpumalanga, Limpopo).

### Statistical analysis

Analyses were conducted in Stata/SE 18 (StataCorp, College Station, TX). Survey weights were applied throughout the analysis, with province specified as the stratification variable and small area layer as the primary sampling unit, to account for the complex survey design and produce nationally representative estimates. Descriptive statistics were used to summarise sample characteristics and estimate prevalence for the three endogenous variables (psychological distress, physiological non-communicable diseases, disability) using counts and percentages. Prevalence comparisons by exogenous variables used Pearson’s chi-square test. For multivariable analysis a Generalized Structural Equation Model (GSEM) was fitted which modelled each endogenous variable as a binary with a binomial distribution and logit link distribution. The log-odds (coefficients) with 95% confidence intervals and p-values are presented to indicate the direction, strength, and statistical significance of associations.

## Results

### Sample characteristics

The sample consisted of 7,897 participants, with 57.6% being female. Two-thirds of the sample (60.6%) were aged 60–69 years, (65.5%) resided in urban areas, and (65.7%) were Black African. A large majority (83.4%) had ever been married and 86.7% were unemployed. Almost half (46.6%) had only primary or no formal education. Provincially, the largest proportion of participants was from Gauteng (23.0%), followed by KwaZulu-Natal (15.6%) and the Eastern Cape (14.1%, Table 1).

**TABLE 1:** Sociodemographic characteristics of adults aged 60 years and older participating in the South African National HIV Prevalence, Incidence, Behaviour and Communication Survey, South Africa, 2022.

Variables	n	(%)	95% CI
Total	7,897	100	​
Individual sex			
Male	2,754	42.4	40.0–44.8
Female	5,138	57.6	55.2–60.0
Age groups			
60–69	4,779	60.6	57.8–63.3
70–79	2,217	27.8	25.6–30.1
80+	901	11.6	9.7–13.8
Race groups			
Black African	6,434	65.7	61.9–69.4
Other races	1,458	34.3	30.6–38.1
Marital status			
Ever married	6,075	83.4	81.5–85.1
Never married	1,777	16.6	14.9–18.5
Highest education obtained			
Non/Primary school	4,491	46.6	43.4–49.8
Secondary school	2,555	37.8	35.2–40.4
Tertiary education	727	15.7	13.3–18.4
Employment status			
Employed	678	13.3	11.1–15.7
Unemployed	6,632	86.7	84.3–88.9
Locality type			
Urban areas	4,685	65.5	61.8–69.1
Rural informal (tribal areas)	2,566	30.0	26.6–33.5
Rural formal (farm areas)	646	4.5	3.2–6.3
Province			
Western Cape	449	17.1	14.7–19.8
Eastern Cape	1,521	14.1	12.3–16.2
Northern Cape	342	2.6	1.9–3.5
Free state	739	4.1	3.4–5.0
KwaZulu-Natal	2,398	15.6	13.6–17.8
North-West	616	5.4	4.5–6.6
Gauteng	682	23.0	19.9–26.5
Mpumalanga	626	5.6	4.4–7.0
Limpopo	524	12.4	10.2–15.0

The number of participants reported for individual independent variables does not always reach the total sample of 7,897 due to missing observations. Abbreviations: CI, confidence interval; NCD, non-communicable disease. Other races: White, Coloured, and Indian/Asian.

### Prevalence of psychological distress, non-communicable diseases, and disability

Among adults aged 60 years and older, the prevalence of psychological distress was 3.1%, physiological NCDs were 59.7%, and disability was 64.1% (Table 2). Women reported a significantly higher prevalence of both disability (69.8% vs. 56.4%) and NCD (65.8% vs. 51.4%) compared to men. Adults aged 70–79 and 80+ years showed significantly higher levels of disability at 72.6% among both male and females, and NCDs (70.1% and 65.3% for women and men respectively) compared to those aged 60–69 years. Black Africans had a significantly higher psychological distress (3.8%) and higher disability (66.8%) compared to other race groups, who reported lower distress (1.8%) and lower disability (59.3%) but a higher prevalence of NCDs (68.7% vs. 54.7%). NCD prevalence was significantly higher in the ever-married group vs. those who had never married (61.5% vs. 51.6%).

**TABLE 2:** Prevalence of psychological distress, non-communicable diseases, and disability among adults aged 60 years and older by sociodemographic characteristics, South Africa, 2022.

Variables	Total	Presence of psychological distress			Total	Presence of non-communicable disease			Total	Presence of disability		
​	n	Row %	95% CI	p-value	n	Row %	95% CI	p-value	n	Row %	95% CI	p-value
Total	7,531	3.1	2.5–3.8	​	7,897	59.7	56.9–62.4	​	7,752	64.1	61.4–66.7	​
Non-communicable disease	​	​	​	0.126	​	​	​	​	​	​	​	​
No	3,060	2.6	1.9–3.5	​	​	​	​	​	​	​	​	​
Yes	4,471	3.4	2.7–4.3	​	​	​	​	​	​	​	​	​
Disability	​	​	​	0.001	​	​	​	<0.001	​	​	​	​
No	2,400	1.9	1.3–2.7	​	2,498	50.6	45.6–55.6	​	​	​	​	​
Yes	5,007	3.8	3.0–4.7	​	5,254	65.1	62.1–68.1	​	​	​	​	​
Individual sex	​	​	​	0.130	​	​	​	<0.001	​	​	​	<0.001
Male	2,614	2.6	1.9–3.6	​	2,754	51.4	46.7–56.0	​	2,696	56.4	51.6–61.0	​
Female	4,913	3.4	2.7–4.3	​	5,138	65.8	63.0–68.6	​	5,051	69.8	67.1–72.3	​
Age groups	​	​	​	0.544	​	​	​	<0.001	​	​	​	0.002
60–69	4,569	2.9	2.3–3.7	​	4,779	53.9	50.6–57.1	​	4,686	58.6	55.7–61.4	​
70–79	2,111	3.6	2.6–4.9	​	2,217	70.1	66.2–73.7	​	2,177	72.6	67.9–76.9	​
80+	851	2.9	1.7–4.9	​	901	65.3	55.2–74.2	​	889	72.6	58.1–83.5	​
Race group	​	​	​	0.003	​	​	​	<0.001	​	​	​	0.012
Black African	6,122	3.8	3.1–4.7	​	6,434	54.7	52.0–57.4	​	6,322	66.8	64.5–69.0	​
Other races	1,404	1.8	1.1–2.8	​	1,458	68.7	62.6–74.1	​	1,425	59.3	53.5–64.7	​
Marital status	​	​	​	0.475	​	​	​	0.001	​	​	​	0.769
Ever married	5,798	3.0	2.4–3.7	​	6,075	61.5	58.4–64.4	​	5,978	64.0	60.9–67.0	​
Never married	1,705	3.5	2.4–5.2	​	1,777	51.6	46.0–57.2	​	1,758	64.9	59.8–69.6	​
Highest education obtained	​	​	​	0.011	​	​	​	0.035	​	​	​	<0.001
Non/Primary school	4,281	4.1	3.2–5.1	​	4,491	56.0	52.7–59.2	​	4,431	71.4	68.9–73.7	​
Secondary school	2,448	2.1	1.5–2.9	​	2,555	64.0	59.8–67.9	​	2,510	59.1	54.6–63.6	​
Tertiary education	700	2.5	1.3–4.8	​	727	61.6	52.7–69.7	​	719	54.1	45.6–62.5	​
Employment status	​	​	​	0.694	​	​	​	0.001	​	​	​	0.018
Employed	651	2.8	1.4–5.4	​	678	45.2	36.4–54.2	​	661	53.6	44.1–62.8	​
Unemployed	6,337	3.2	2.6–3.9	​	6,632	61.1	58.2–63.9	​	6,540	65.3	62.3–68.1	​
Locality type	​	​	​	0.490	​	​	​	<0.001	​	​	​	0.083
Urban areas	4,493	3.0	2.3–3.8	​	4,685	63.5	59.6–67.1	​	4,580	62.4	58.7–66.0	​
Rural informal (tribal areas)	2,421	3.5	2.5–5.0	​	2,566	52.0	48.3–55.6	​	2,537	67.9	64.6–71.1	​
Rural formal (farm areas)	617	2.1	0.9–5.1	​	646	56.3	44.4–67.5	​	635	63.1	52.4–72.6	​
Province	​	​	​	<0.001	​	​	​	0.001	​	​	​	0.002
Western Cape	436	2.2	1.1–4.3	​	449	70.2	60.6–78.3	​	437	60.8	53.1–68.0	​
Eastern Cape	1,428	6.1	4.2–8.7	​	1,521	59.2	54.8–63.4	​	1,495	72.6	68.0–76.8	​
Northern Cape	326	4.0	1.7–9.3	​	342	59.3	44.9–72.2	​	335	69.3	60.9–76.5	​
Free state	707	2.9	1.9–4.5	​	739	63.8	57.4–69.6	​	728	68.3	63.2–73.1	​
KwaZulu-Natal	2,337	3.4	2.4–4.9	​	2,398	56.9	52.5–61.2	​	2,376	70.9	67.0–74.6	​
North-West	587	1.9	1.0–3.7	​	616	63.8	58.9–68.4	​	606	63.6	57.7–69.1	​
Gauteng	653	2.5	1.5–4.1	​	682	61.6	53.2–69.4	​	650	59.8	51.6–67.6	​
Mpumalanga	596	6.4	3.5–11.4	​	626	44.8	39.0–50.8	​	608	50.9	42.7–59.0	​

Furthermore, Table 2 also shows that those with no or only primary schooling had the highest psychological distress (4.1%) and the highest disability (71.4%). Adults with secondary schooling showed the highest NCD prevalence (64.0%), while tertiary-educated adults reported moderate disability (54.1%) and higher NCD levels (61.6%). Unemployed adults had significantly higher disability (65.3%) and NCDs (61.1%) compared those who were employed. Urban area residents had a significantly higher NCD prevalence (63.5%) compared to Rural formal and Rural informal at 56.3% and 52.0%, respectively. Older adults with NCDs reported higher levels of psychological distress (3.4% vs. 2.6%). Mpumalanga and Eastern Cape showed the highest prevalence of psychological distress (6.4% and 6.1%), while Western Cape had the highest NCD prevalence (70.2%). Disability was most common in Eastern Cape (72.6%) and KwaZulu-Natal (70.9%).

### Factors associated with psychological distress, non-communicable diseases, and disability

Figure 1 illustrates the conceptual framework fitted using a generalized structural equation model. Age, education, employment, locality, sex, and race were included as exogenous variables, with direct and indirect paths to non-communicable diseases (NCDs), disability, and psychological distress. Parameter estimates are presented in the table that follows.

**FIGURE 1:**
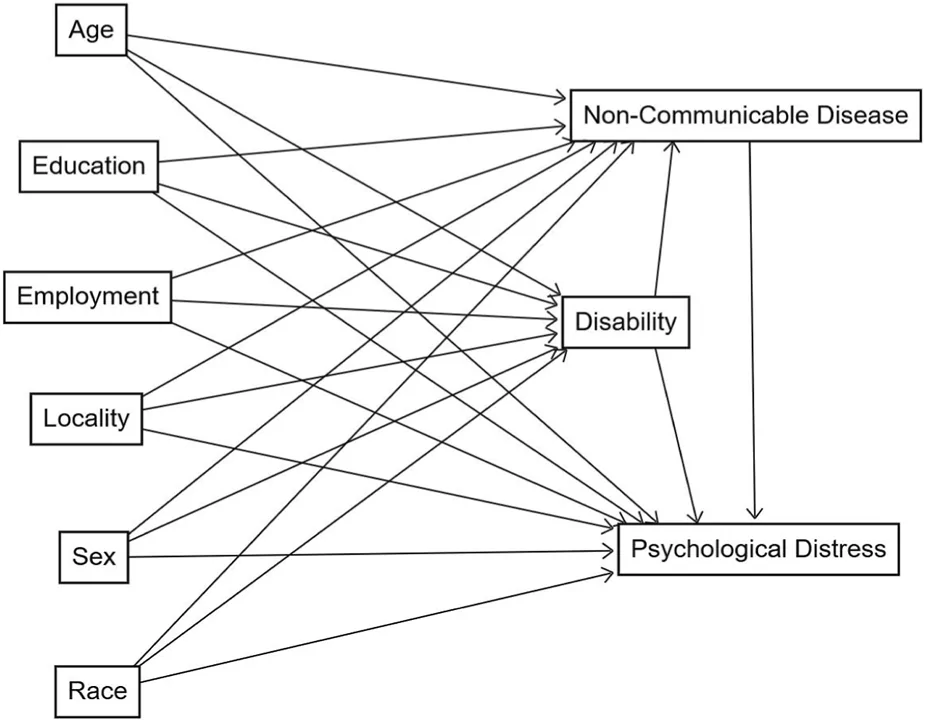
Generalized Structural Equation Model (direct pathways) of factors associated with psychological distress Non-Communicable Diseases, and Disability.

Table 3 shows the parameter estimates from the Generalized Structural Equation Model of factors associated with psychological distress, NCDs, and disability. The final GSEM analysis included 7,101 participants, with less than 10% missing information in the model. Older adults with at least one NCD had significantly higher odds of reporting psychological distress compared to those without NCDs (log-odds = 0.43, p = 0.028). Participants with disabilities had higher odds of NCD (log-odds = 0.53, p < 0.001) compared to those without disabilities. Women did not differ from men regarding psychological distress, but they had significantly higher odds of reporting NCDs (log-odds = 0.70, p < 0.001) and disability (log-odds = 0.51, p < 0.001). Adults aged 70–79 years had higher odds of both NCDs (log-odds = 0.53, p < 0.001) and disability (log-odds = 0.55, p < 0.001) compared to those aged 60–69 years. Participants from Other racial groups had significantly lower odds of psychological distress (log-odds = −0.84, p = 0.008) but higher odds of NCDs (log-odds = 0.53, p = 0.001) compared to Black Africans.

**TABLE 3:** Parameter Estimates from the Generalized Structural Equation Model of actors associated with psychological distress, non-Communicable diseases, and disability.

Variables	Presence of psychological distress				Presence of non-communicable disease				Presence of disability			
​	Log-odds	95% CI		p-value	Log-odds	95% CI		p-value	Log-odds	95% CI		p-value
Non-communicable disease												
No	Ref	​	​	​	​	​	​	​	​	​	​	​
Yes	0.43	0.05	0.82	0.028	​	​	​	​	​	​	​	​
Disability												
No	Ref	​	​	​	Ref	​	​	​	​	​	​	​
Yes	0.42	−0.02	0.87	0.064	0.53	0.31	0.75	<0.001	​	​	​	​
Individual sex												
Male	Ref	​	​	​	Ref	​	​	​	Ref	​	​	​
Female	0.03	−0.36	0.42	0.875	0.70	0.50	0.90	<0.001	0.51	0.28	0.74	<0.001
Age groups												
60–69	​	​	​	​	​	​	​	​	​	​	​	​
70–79	0.21	−0.22	0.64	0.336	0.53	0.29	0.77	<0.001	0.55	0.28	0.82	<0.001
80+	0.00	−0.64	0.65	0.990	0.44	−0.01	0.88	0.057	0.40	−0.18	0.98	0.178
Race group												
Black African	Ref	​	​	​	Ref	​	​	​	Ref	​	​	​
Other races	−0.84	−1.46	−0.22	0.008	0.53	0.21	0.86	0.001	−0.10	−0.39	0.19	0.503
Marital status												
Ever married	Ref	​	​	​	Ref	​	​	​	Ref	​	​	​
Never married	0.16	−0.32	0.63	0.514	−0.35	−0.59	−0.10	0.005	−0.06	−0.33	0.20	0.647
Highest education obtained												
Non/Primary school	Ref	​	​	​	Ref	​	​	​	Ref	​	​	​
Secondary school	−0.61	−1.08	−0.14	0.010	0.26	0.03	0.49	0.027	−0.42	−0.65	−0.18	<0.001
Tertiary education	0.05	−0.63	0.73	0.885	−0.06	−0.46	0.34	0.768	−0.72	−1.13	−0.32	<0.001
Employment status												
Employed	Ref	​	​	​	Ref	​	​	​	Ref	​	​	​
Unemployed	−0.30	−0.97	0.38	0.388	0.57	0.16	0.98	0.006	0.05	−0.36	0.46	0.805
Locality type												
Urban areas	Ref	​	​	​	Ref	​	​	​	Ref	​	​	​
Rural informal (tribal areas)	−0.16	−0.64	0.32	0.503	−0.40	−0.63	−0.17	0.001	−0.08	−0.30	0.14	0.487
Rural formal (farm areas)	−0.34	−1.25	0.57	0.459	−0.10	−0.53	0.32	0.639	0.01	−0.51	0.53	0.974

Older adults who were never-married did not differ with regards to psychological distress or disability but had lower odds of reporting NCDs (log-odds = −0.35, p = 0.005) than those who were ever married. Having a secondary education was associated with lower odds of psychological distress (log-odds = −0.61, p = 0.010) and disability (log-odds = −0.42, p < 0.001), but slightly higher odds of reporting NCDs (log-odds = 0.26, p = 0.027), relative to primary or no schooling. Tertiary education showed strong protective effects against disability (log-odds = −0.72, p < 0.001), with no significant associations for distress or NCDs. Unemployed older adults were more likely to report NCDs (log-odds = 0.57, p = 0.006), with no significant differences in reported psychological distress or disability compared to the employed. Living in a rural informal area was associated with lower odds of reporting NCDs (log-odds = −0.40, p = 0.001) but showed no association with psychological distress or disability when compared to urban residents. There were no significant associations across outcomes among rural formal residence (Table 3).

## Discussion

This study aimed to assess the burden and socio-demographic patterning of non-communicable diseases, disability and psychological distress among older South Africans, and to examine the social determinants and interrelationships of these outcomes using a Generalized Structural Equation Model. In this study, the prevalence of psychological distress, NCDs and disability among adults aged 60 years and older was 3.1%, 59.7% and 64.1%, respectively. Although the K10 has demonstrated its ability to discriminate levels of nonspecific psychological distress in South African populations (Andersen et al., 2011; Chamane et al., 2026; Hoffman et al., 2022), it has not been specifically validated among adults aged 60 years and older in the South African setting. Consequently, its ability to identify psychological distress in this population may be limited, and the observed prevalence may underestimate the true burden. Furthermore, under recognition and under reporting of mental illness among older adults, potentially driven by stigma, generational attitudes towards mental health, the overlap of psychological symptoms with physical conditions, and limited recognition of mental health problems in routine healthcare (Adams et al., 2024; Elshaikh et al., 2023). The final model revealed that older adults with at least one NCD had significantly higher odds of experiencing psychological distress, while those with disabilities were more likely to have an NCD. These findings are consistent with previous research showing that NCDs increase the risk of mental health challenges, and that functional limitations often accompany multiple chronic diseases (Cini et al., 2024; Mehari et al., 2023; Sharma et al., 2021). These findings point to a need for integrated approaches that treat physical, mental and functional health collectively rather than in isolation.

As observed in other studies, women and those aged 70–79 years had higher odds of having both NCDs and disability compared to men and adults aged 60–69 years. These findings align with evidence showing that ageing and sex shape vulnerability to chronic health conditions and functional limitations (Agyekum et al., 2024; Wandera et al., 2014; 2015). The study confirms the disproportionate burden of NCDs and disability among women and older adults, reflecting patterns observed in other populations (Zungu et al., 2019). The WHO attributes the disparity in NCD risk between men and women to both biological and behavioural factors (WHO, 2020). Other researchers further highlight the role of obesity, particularly its higher prevalence among women, as a major driver of NCDs (Ejigu and Tiruneh, 2023; Ferreira and Vincent-Lambert, 2025; Mendham et al., 2021). The findings point to the importance of differentiated approaches that factor demographics and associated risks when addressing physical and functional health. Targeted, age-sensitive interventions could be prioritised to improve health outcomes among high-risk populations. In particular, policymakers and programme implementers could invest in community-based strategies that integrate chronic disease management, functional support, and mental health services. Such integrated approaches could help address the complex and overlapping health needs of vulnerable groups, particularly older adults, and could enhance overall wellbeing and quality of life.

Similar to previous research, participants from other racial groups had significantly lower odds of psychological distress compared to Black Africans (Harriman et al., 2022; Ramlagan et al., 2025). Previous studies have demonstrated that racial disparities in psychological distress within South Africa are associated with socio-economic and structural inequalities, which increase susceptibility to such distress (Ardington and Case, 2010; Harriman et al., 2022). While Black African populations generally face higher rates of psychological distress, recent evidence indicates that these racial differences predominantly manifest at lower symptom levels, with disparities considerably diminishing in severe cases (Posel and Oyenubi, 2025). This implies that additional factors could be considered when analysing the relationship between race and psychological distress across all age groups.

Similar to previous research, participants from racial groups other than Black African had significantly lower odds of psychological distress (Harriman et al., 2022; Ramlagan et al., 2025). Previous studies suggest that racial disparities in psychological distress in South Africa are associated with socioeconomic and structural inequalities that shape exposure to adverse living conditions and psychosocial stressors (Ardington and Case, 2010; Harriman et al., 2022). Recent evidence further indicates that racial differences may be more pronounced at lower symptom levels and diminish in cases of severe psychological distress (Posel and Oyenubi, 2025). These findings suggest that the relationship between race and psychological distress is complex and may reflect differences in socioeconomic circumstances and broader structural determinants of health.

Contrary to national data from Statistics South Africa (Stats SA) show that Black Africans have the highest mortality related to NCDs, this study found that they were less likely to report having a physiological NCD compared to other population groups (Statistics South Africa, 2023). This apparent discrepancy may reflect differences in diagnosis, access to healthcare, health information, and self-reporting of chronic conditions. Differences in socioeconomic circumstances, educational attainment, and health-seeking behaviours may also contribute to variation in reported NCD prevalence across population groups. However, these explanations were not directly examined in the present study and should therefore be interpreted cautiously. These findings highlight the need to consider racial disparities in the reporting and detection of NCDs among older adults in South Africa. Improving equitable access to screening, diagnosis, and ongoing healthcare may help address potential gaps in the identification and management of chronic conditions across population groups.

Consistent with studies linking education to later-life health positive outcomes (Kunda et al., 2025; Roomaney et al., 2022), those with secondary education had reduced odds of having psychological distress and disability but slightly higher odds of NCDs compared to those with only primary education. Having a tertiary education showed protective effects only for disability. The observed associations between education levels and health outcomes among older South Africans aligns with existing literature highlighting the complex relationship between educational attainment and health in later life (Kunda et al., 2025). This pattern suggests that secondary education may provide individuals with better coping mechanisms, access to resources, and health literacy, which contribute to improved mental health and functional status. However, the slightly higher odds of NCDs among this group could be attributed to lifestyle factors or increased healthcare access leading to higher detection rates. Tertiary education demonstrated protective effects solely for disability, which may reflect the benefits of higher education in promoting healthier lifestyles and better access to healthcare services including screening and preventive measures. These findings underscore the significant role of education as a determinant of health outcomes in older adults. They highlight the importance of considering educational attainment when developing public health strategies aimed at reducing health disparities among the elderly. Addressing educational inequalities could be pivotal in improving health outcomes and quality of life for older populations.

Finally, research has also pointed out the role of employment and residence in shaping chronic disease risk (Li et al., 2025; Silvaggi et al., 2020; Yildiz et al., 2021). In this study, unemployment was associated with higher odds of NCDs, while residing in rural informal areas and never having been married were linked to lower odds of NCDs compared to being employed, living in urban areas and ever being married, respectively. The association between unemployment and increased odds of NCDs among older South Africans aligns with findings from the National Income Dynamics Study, which indicate that unemployment is a significant risk factor for chronic diseases in this population and notes variation by sex (Lawana et al., 2023). This relationship may be attributed to factors such as limited access to healthcare, increased stress levels, and reduced opportunities for physical activity, all of which can contribute to the development and progression of NCDs. Conversely, residing in rural informal areas and never having been married were associated with lower odds of NCDs compared to being employed, living in urban areas, and ever being married, respectively. These findings are consistent with studies that have observed lower prevalence rates of chronic diseases in rural settings, possibly due to lifestyle factors such as increased physical activity and dietary habits (Ajaero et al., 2022). Additionally, the lower odds of NCDs among individuals who have never been married may reflect differences in health behaviours and social support systems, which can influence health outcomes (Ali et al., 2024). These results show the complex interplay between employment status, residential location, marital status, and chronic disease risk. They highlight the importance of considering these socio-demographic factors when developing public health strategies aimed at reducing the burden of NCDs among older adults in South Africa. Addressing the underlying social determinants of health, such as improving access to healthcare, promoting healthy lifestyles, and enhancing social support networks, may be crucial in mitigating the risk of NCDs in this population.

### Strengths and limitations

The study provides nationally representative insights into the prevalence and social determinants of multimorbidity among older South Africans. Furthermore, the use of a large, population-based dataset with appropriate sampling weights, allowing for generalisable estimates at the national level. In addition, the use of established and validated instruments, such as the Kessler Psychological Distress Scale (K10), enhances the reliability and comparability of the psychological distress measure. Despite this, the study has several limitations. The cross-sectional design prevents causal inference, thus only presents the associations between psychological distress, non-communicable diseases, disability, and the socio-demographic factors. Aggregating distinct racial groups such as White, Coloured, Asian/Indian, and others into a single ‘Other’ category may obscure the true prevalence of non-communicable diseases (NCDs) and disabilities within these populations, thereby limiting our ability to track, accurately understand and address group-specific health disparities and associated risk factors in the future. The self-reported nature of the data may result in an underestimation of psychological NCD prevalence, particularly among undiagnosed individuals. The GSEM implementation used in Stata does not provide a conventional global model-fit statistic for the fitted model; therefore, model fit was not reported as a separate diagnostic. We acknowledge that the implementation of survey-adjusted GSEM is software-dependent, and estimates and particularly variance estimates may differ across statistical software packages because of differences in the algorithms and procedures used to incorporate complex survey features into GSEM. The joint modelling does not automatically eliminate omitted-variable or simultaneity bias, and that the interpretation of these relationships depends on the model specification and assumptions.

## Conclusion

This study identified cross-sectional associations between psychological distress, non-communicable diseases (NCDs) and disability among older adults in South Africa, alongside variations across socio-demographic factors. The findings suggest that differentiated, community-based primary health programmes combining chronic disease management, mental health support and social services may be beneficial for groups experiencing a greater burden of these conditions. Having at least one NCD was associated with a higher likelihood of psychological distress, while the presence of a disability was associated with a higher likelihood of having an NCD. These associations should be interpreted cautiously because the cross-sectional design does not establish temporal or causal relationships, and potential overlap in the operationalisation of the binary outcomes may influence the observed associations. Nevertheless, the findings highlight the importance of considering physical health, psychological wellbeing and disability when planning services for older adults. Social policies that address structural factors affecting the ageing population remain important. Strengthening prevention, early detection, community support networks and coordinated management of multimorbidity may help improve health and wellbeing among older adults and reduce the long-term burden on the health system in South Africa.

## Data Availability

The raw data supporting the conclusions of this article will be made available by the authors, without undue reservation.

## References

[B1] Adams C. Gringart E. Strobel N. (2024). Barriers to mental health help-seeking among older adults with chronic diseases. Aust. Psychol. 59 (2), 154–165. 10.1080/00050067.2023.2282540

[B2] Agyekum M. W. Afrifa-Anane G. F. Kyei-Arthur F. (2024). Prevalence and correlates of disability in older adults, Ghana: evidence from the Ghana 2021 population and housing census. BMC Geriatr. 24 (1), 52. 10.1186/s12877-023-04587-6 PMC10785330 38212686

[B3] Aitken R. J. Norman R. J. (2025). “Epidemiology of falling fertility: the contribution of social, environmental, and genetic forces,” in Fertility and Sterility. 10.1016/j.fertnstert.2025.04.048 40348158

[B4] Ajaero C. K. De Wet N. Odimegwu C. O. (2022). Integrating rural–urban differentials in the appraisal of prevalence and risk factors of non-communicable diseases in South Africa. GeoJournal 87 (2), 829–843. 10.1007/s10708-020-10282-5

[B5] Ali M. A. Ali A. O. Abokor A. H. Farih O. A. Muse A. H. (2024). Prevalence and determinants of non-communicable diseases among child-bearing women in Somaliland from a 2020 nationwide survey in Somaliland. Discov. Public Health 21 (1), 229. 10.1186/s12982-024-00371-y

[B6] Andersen L. S. Grimsrud A. Myer L. Williams D. R. Stein D. J. Seedat S. (2011). The psychometric properties of the K10 and K6 scales in screening for mood and anxiety disorders in the South African stress and health study. Int. J. Methods Psychiatric Res. 20 (4), 215–223. 10.1002/mpr.351 22113964 PMC6878543

[B7] Ardington C. Case A. (2010). Interactions between mental health and socioeconomic status in the South African national income dynamics study. J. Stud. Econ. Econ. 34 (3), 69–85. 10.1080/10800379.2010.12097210 PMC3170771 21915159

[B8] Basu D. (2022). National strategic plan for the prevention and control of non-communicable diseases in South Africa. South. Afr. J. pub. Health 5 67. 10.7196/sajph.2022.v5.i3.174

[B9] Belayneh A. Chelkeba L. Amare F. Fisseha H. Abdissa S. G. Kaba M. (2024). Investigation of non-communicable diseases prevalence, patterns, and patient outcomes in hospitalized populations: a prospective observational study in three tertiary hospitals. J. Health, Popul. Nutr. 43 (1), 128. 10.1186/s41043-024-00599-z PMC11337899 39164738

[B10] Bredenkamp C. Burger R. Jourdan A. Van Doorslaer E. (2021). Changing inequalities in health-adjusted life expectancy by income and race in South Africa. Health Syst. Reform 7 (2), e1909303. 10.1080/23288604.2021.1909303 34402377

[B11] Carmel S. (2019). Health and well-being in late life: gender differences worldwide. Front. Med. 6, 218. 10.3389/fmed.2019.00218 31649931 PMC6795677

[B12] Chamane S. Mabaso M. Sewpaul R. Makola L. Jooste S. Moyo S. (2026). Psychometric evaluation of the kessler 10 measure of psychosocial distress using the 2022 South African population-based household survey. Curr. Psychol. 45 (10), 1021. 10.1007/s12144-026-09563-y PMC13222254 42227010

[B13] Chang A. Y. Gómez-Olivé F. X. Payne C. Rohr J. K. Manne-Goehler J. Wade A. N. (2019). Chronic multimorbidity among older adults in rural South Africa. BMJ Glob. Health 4 (4), e001386. 10.1136/bmjgh-2018-001386 31423345 PMC6688670

[B14] Cini K. I. Dumuid D. Francis K. L. Wulan N. R. Sawyer S. M. Handy Agung F. (2024). The relationship between non-communicable disease risk and mental wellbeing in adolescence: a cross-sectional study utilising objective measures in Indonesia. BMC Public Health 24 (1), 3416. 10.1186/s12889-024-20902-1 PMC11653778 39695503

[B15] Doan T. Shin W. Mehta N. (2022). To what extent were life expectancy gains in South Africa attributable to declines in HIV/AIDS mortality from 2006 to 2017? A life table analysis of age-specific mortality. Demogr. Res. 46, 547–564. 10.4054/demres.2022.46.18

[B16] Ejigu B. A. Tiruneh F. N. (2023). The link between overweight/obesity and noncommunicable diseases in Ethiopia: evidences from nationwide WHO STEPS survey 2015. Int. J. Hypertens. 2023 (1), 2199853. 10.1155/2023/2199853 PMC10667048 38023617

[B17] Elshaikh U. Sheik R. Saeed R. K. M. Chivese T. Alsayed Hassan D. (2023). Barriers and facilitators of older adults for professional mental health help-seeking: a systematic review. BMC Geriatr. 23 (1), 516. 10.1186/s12877-023-04229-x PMC10463345 37626290

[B18] Everard C. Vuik S. Lerouge A. Cecchini M. (2025). “Exploring the Relationship between Non-Communicable Diseases and Depression”. in OECD Health Working Papers. 178th ed. Paris, France: Organisation for Economic Co-Operation and Development.

[B19] Ferreira S. Vincent-Lambert C. (2025). Prevalence of obesity among adults in otjomuise community in Namibia. Health SA Gesondheid 30, 3074. 10.4102/hsag.v30i0.3074 PMC12421579 40936641

[B20] Gu D. Andreev K. Dupre M. E. (2021). Major trends in population growth around the world. China CDC Wkly. 3 (28), 604–613. 10.46234/ccdcw2021.160 PMC8393076 34594946

[B21] Hamasaki H. (2025). The relationship between socioeconomic status and health behaviors in older adults: a narrative review. Healthcare 13 (14), 1669. 10.3390/healthcare13141669 PMC12295311 40724694

[B22] Harriman N. W. Williams D. R. Morgan J. W. Sewpaul R. Manyaapelo T. Sifunda S. (2022). Racial disparities in psychological distress in post-apartheid South Africa: results from the SANHANES-1 survey. Soc. Psychiatry Psychiatric Epidemiol. 57 (4), 843–857. 10.1007/s00127-021-02175-w PMC8494453 34617128

[B23] Hoffman J. Cossie Q. Ametaj A. A. Kim H. H. James R. Stroud R. E. (2022). Construct validity and factor structure of the Kessler-10 in South Africa. BMC Psychol. 10 (1), 177. 10.1186/s40359-022-00883-9 PMC9290237 35851071

[B24] Ida S. Kaneko R. Imataka K. Okubo K. Shirakura Y. Azuma K. (2020). Factors associated with social isolation and being homebound among older patients with diabetes: a cross-sectional study. BMJ Open 10 (11), e037528. 10.1136/bmjopen-2020-037528 33154045 PMC7646328

[B25] Jaul E. Barron J. (2017). Age-related diseases and clinical and public health implications for the 85 years old and over population. Front. Public Health 5, 335. 10.3389/fpubh.2017.00335 29312916 PMC5732407

[B26] Khalili G. Zargoush M. Huang K. Ghazalbash S. (2024). Exploring trajectories of functional decline and recovery among older adults: a data-driven approach. Sci. Rep. 14 (1), 6340. 10.1038/s41598-024-56606-0 PMC10943109 38491130

[B27] Khan H. T. A. Addo K. M. Findlay H. (2024). Public health challenges and responses to the growing ageing populations. Public Health Challenges 3 (3), e213. 10.1002/puh2.213 PMC12039680 40496520

[B28] Kunda A. C. Nshimyiryo A. Kuodi P. Hagenimana P. Dusabayezu S. Ngwakongnwi E. (2025). Prevalence and risk factors of anxiety and depression among non-communicable diseases clinic attendees in rural Rwanda: a cross-sectional study. BMJ Open 15 (7), e102829. 10.1136/bmjopen-2025-102829 PMC12278158 40685237

[B29] Lawana N. Kapingura F. M. Tsegaye A. (2023). The impact of non-communicable diseases on employment status in South Africa. Cogent Econ. Finance 11 (2), 2246005. 10.1080/23322039.2023.2246005

[B30] Li W. Peng C. Luo W. Chen X. Zeng Q. Kang B. (2025). Residential environment and risk of chronic diseases: a prospective study. Ecotoxicol. Environ. Saf. 295, 118141. 10.1016/j.ecoenv.2025.118141 40187210

[B31] Mehari E. A. Kidane R. B. Areki M. F. Seid A. M. Gelaye A. T. (2023). The magnitude and associated factors of anxiety and depression among non-communicable chronic disease patients during COVID-19 pandemic in a resource-limited setting. Clin. Epidemiol. Glob. Health 21, 101274. 10.1016/j.cegh.2023.101274 PMC10028352 37033721

[B32] Mendham A. E. Goedecke J. H. Micklesfield L. K. Brooks N. E. Faber M. Christensen D. L. (2021). Understanding factors associated with sarcopenic obesity in older African women from a low-income setting: a cross-sectional analysis. BMC Geriatr. 21 (1), 247. 10.1186/s12877-021-02132-x 33853546 PMC8048063

[B33] Navaneetham K. Arunachalam D. (2025). “Global population aging, 1950-2050,” in Handbook of Aging, Health and Public Policy: Perspectives from Asia. 10.1007/978-981-99-7842-7_154

[B34] Peiris D. Feyer A.-M. Barnard J. Billot L. Bouckley T. Campain A. (2024). Overcoming silos in health care systems through meso-level organisations–a case study of health reforms in New South Wales, Australia. Lancet Regional Health–Western Pac. 44, 101013. 10.1016/j.lanwpc.2024.101013 PMC10879775 38384947

[B35] Posel D. Oyenubi A. (2025). Divergence in the size and composition of the race gap in mental health: evidence from South Africa. SSM - Popul. Health 31, 101829. 10.1016/j.ssmph.2025.101829 PMC12221280 40607176

[B36] Ramlagan S. Naidoo I. North A. Jooste S. (2025). Psychological distress in South Africa: analysis of 2017 national household survey data. Health SA Gesondheid 30, 2920. 10.4102/hsag.v30i0.2920 41069565 PMC12505445

[B37] Rasesemola R. M. Mmusi-Phetoe R. M. Havenga Y. (2023). Social determinants of health in non-communicable diseases prevention policies in South Africa. Curationis 46 (1), 1–8. 10.4102/curationis.v46i1.2387 PMC10476442 37782234

[B38] Roomaney R. A. Van Wyk B. Cois A. Pillay-van Wyk V. (2022). Inequity in the distribution of non-communicable disease multimorbidity in adults in South Africa: an analysis of prevalence and patterns. Int. J. Public Health 67, 1605072. 10.3389/ijph.2022.1605072 PMC9426027 36051505

[B39] Rudnicka E. Napierała P. Podfigurna A. Męczekalski B. Smolarczyk R. Grymowicz M. (2020). The world health organization (WHO) approach to healthy ageing. Maturitas 139, 6–11. 10.1016/j.maturitas.2020.05.018 32747042 PMC7250103

[B40] Salazar L. J. Hegde D. Srinivasan K. Heylen E. Ekstrand M. L. (2025). Correlates of disability among primary care patients with common mental disorders and chronic medical conditions-a cross-sectional study from rural South India. Soc. Psychiatry Psychiatric Epidemiol. 60 (4), 859–868. 10.1007/s00127-024-02727-w 39044019 PMC11754534

[B41] Sharma P. Maurya P. Muhammad T. (2021). Number of chronic conditions and associated functional limitations among older adults: cross-sectional findings from the longitudinal aging study in India. BMC Geriatr. 21 (1), 664. 10.1186/s12877-021-02620-0 34814856 PMC8609791

[B42] Silvaggi F. Eigenmann M. Scaratti C. Guastafierro E. Toppo C. Lindstrom J. (2020). Employment and chronic diseases: suggested actions for the implementation of inclusive policies for the participation of people with chronic diseases in the labour market. Int. J. Environ. Res. Public Health 17 (3), 820. 10.3390/ijerph17030820 32013004 PMC7037802

[B43] Solanki G. Kelly G. Cornell J. Daviaud E. Geffen L. (2019). Population ageing in South Africa: trends, impact, and challenges for the health sector. South Afr. Health Rev. 2019 (1), 175–182. PMC8525465 34671174

[B44] Summers A. Leidman E. Pereira Figueira Periquito I. M. Bilukha O. O. (2019). Serious psychological distress and disability among older persons living in conflict affected areas in eastern Ukraine: a cluster-randomized cross-sectional household survey. Confl. Health 13 (1), 10. 10.1186/s13031-019-0194-0 31080498 PMC6503356

[B45] Tomita A. Burns J. K. (2013). Depression, disability and functional status among community-dwelling older adults in South Africa: evidence from the first South African national income dynamics study. Int. J. Geriatric Psychiatry 28 (12), 1270–1279. 10.1002/gps.3954 23512338 PMC3751982

[B46] Trudel-Fitzgerald C. Millstein R. A. Von Hippel C. Howe C. J. Tomasso L. P. Wagner G. R. (2019). Psychological well-being as part of the public health debate? Insight into dimensions, interventions, and policy. BMC Public Health 19 (1), 1712. 10.1186/s12889-019-8029-x 31856772 PMC6923969

[B47] Wandera S. O. Ntozi J. Kwagala B. (2014). Prevalence and correlates of disability among older ugandans: evidence from the Uganda national household survey. Glob. Health Action 7 (1), 25686. 10.3402/gha.v7.25686 25413721 PMC4238972

[B48] Wandera S. O. Kwagala B. Ntozi J. (2015). Prevalence and risk factors for self-reported non-communicable diseases among older ugandans: a cross-sectional study. Glob. Health Action 8 (1), 27923. 10.3402/gha.v8.27923 26205363 PMC4513181

[B49] Waterhouse P. Van Der Wielen N. Banda P. C. Channon A. A. (2017). The impact of multi-morbidity on disability among older adults in South Africa: do hypertension and socio-demographic characteristics matter? Int. J. Equity Health 16 (1), 62. 10.1186/s12939-017-0537-7 28388911 PMC5385014

[B50] WHO (2020). UN Decade of Health Ageing: Plan of Action 2021-2012. Available online at: https://cdn.who.int/media/docs/default-source/decade-of-healthy-ageing/decade-proposal-final-apr2020-en.pdf (Accessed July 29, 2026).

[B51] WHO (2025a). Ageing and Health. Available online at: https://www.who.int/news-room/fact-sheets/detail/ageing-and-health (Accessed July 29, 2026).

[B52] WHO (2025b). Ageing: Global Population. Available online at: https://www.who.int/news-room/questions-and-answers/item/population-ageing (Accessed July 29, 2026).

[B54] Xiao S. Shi L. Dong F. Zheng X. Xue Y. Zhang J. (2022). The impact of chronic diseases on psychological distress among the older adults: the mediating and moderating role of activities of daily living and perceived social support. Aging & Ment. Health 26 (9), 1798–1804. 10.1080/13607863.2021.1947965 34238092

[B55] Yildiz B. Burdorf A. Schuring M. (2021). The influence of chronic diseases and multimorbidity on entering paid employment among unemployed Persons–a longitudinal register-based study. Scand. J. Work, Environ. & Health 47 (3), 208–216. 10.5271/sjweh.3942 PMC8126442 33350454

[B56] Zuma K. Zungu N. P. Moyo S. Marinda E. Simbayi L. C. Jooste S. E. (2025). The Sixth South Africa National HIV, Incidence and Behaviour Survey, 2022: A Summary Report. Cape Town: HSRC Press.

[B57] Zungu N. P. Mabaso M. L. Kumalo F. Sigida S. Mlangeni L. Wabiri N. (2019). Prevalence of non-communicable diseases (NCDs) and associated factors among HIV positive educators: findings from the 2015/6 survey of health of educators in public schools in South Africa. PLoS ONE 14 (2), e0209756. 10.1371/journal.pone.0209756 30735504 PMC6368278

